# Delayed diagnosis of spinal cord schistosomiasis in a non-endemic country: A tertiary referral centre experience

**DOI:** 10.1371/journal.pntd.0009161

**Published:** 2021-02-11

**Authors:** Angus de Wilton, Dinesh Aggarwal, Hans Rolf Jäger, Hadi Manji, Peter L. Chiodini

**Affiliations:** 1 The Hospital for Tropical Diseases, University College London Hospitals, United Kingdom; 2 UCL Queen Square Institute of Neurology, London, United Kingdom; 3 Lysholm Department of Neuroradiology, National Hospital for Neurology and Neurosurgery, University College London Hospitals, London, United Kingdom; 4 Department of Neurology, National Hospital for Neurology and Neurosurgery, University College London Hospitals, London, United Kingdom; 5 The London School of Hygiene and Tropical Medicine, London, United Kingdom; University of the District of Columbia, George Washington University School of Medicine and Health Sciences, UNITED STATES

## Abstract

**Background:**

Neuroschistosomiasis is a severe complication of schistosomiasis, triggered by the local immune reaction to egg deposition, with spinal cord involvement the most well recognised form. Early treatment with praziquantel and high dose steroids leads to a reduction of neurological sequelae. The rarity of this condition in returning travellers to high income countries can result in delayed diagnosis and treatment. We aimed to evaluate the diagnosis and management of neuroschistosomiasis in a UK national referral centre.

**Materials/Methods:**

A retrospective review of confirmed clinical cases of spinal schistosomiasis referred to the Hospital for Tropical Diseases, UK, between January 2016 and January 2020 was undertaken. Electronic referral records were interrogated and patient demographic, clinical, laboratory, and radiological data collected.

**Results:**

Four cases of neuroschistosomiasis were identified. The median age at diagnosis was 28 (range 21 to 50) with three male patients. All patients had epidemiological risk factors for schistosomiasis based on travel history and freshwater exposure; two in Uganda (River Nile), one in Malawi and one in Nigeria. All patients presented with features of transverse myelitis including back pain, leg weakness, paraesthesia and urinary dysfunction. The mean time from presentation to health services to definitive treatment was 42.5 days (range 16–74 days). Diagnosis was confirmed with CSF serology for schistosomiasis in all cases. Radiological features on MRI spine included enhancement focused predominantly in the lower thoracic spinal cord in three cases and the conus in one patient. All patients received a minimum of three days of oral praziquantel and high dose steroids. At three-month follow-up, one patient had complete resolution of symptoms and three had residual deficit; one patient was left with urinary and faecal incontinence, another had urinary retention, and the final patient has persistent leg pains and constipation.

**Conclusion:**

We observed a marked delay in diagnosis of neuroschistosomiasis in a non-endemic country. We advocate undertaking a thorough travel history, early use of imaging and CSF schistosomal serology to ensure early diagnosis of neuroschistosomiasis in patients presenting with consistent symptoms. If schistosomal diagnostics are not immediately available, presumptive treatment under the guidance of a tropical medicine specialist should be considered to minimize the risk of residual disability. We advocate for consensus guidelines to be produced and reporting to be performed in a uniform way for patients with spinal schistosomiasis.

## Author summary

Schistosomiasis is a parasitic neglected tropical disease causing morbidity and mortality in several tropical regions. The most commonly described complications of schistosomiasis include urinary tract and gastrointestinal pathology. However, ectopic migration of parasitic worms and their eggs into the central nervous system can lead to profound and life altering disability. This phenomenon, referred to as ‘Neuroschistosomiasis’ is a rarely reported in non-endemic countries. However, occurrence is increasingly recognised in non-endemic regions due to the increase in global travel. We report the clinical and radiological characteristics of four patients who developed neuroschistosomiasis following tropical freshwater exposure. The report informs diagnosis of schistosomiasis, diagnostic features including subtle radiological findings typical of schistosomiasis, and management of neuroschistosomiasis. The report further highlights the delays in diagnosis of these patients, and the importance of travel history and seeking specialist parasitological advice when patients present with spinal cord syndromes following relevant exposures.

## Introduction

200 million people worldwide are currently estimated to be infected with schistosomiasis [1]; 120 million of these have symptomatic schistosomiasis and 20 million develop severe disease [2,3]. Schistosomiasis is a parasitic infection acquired through contact with contaminated fresh water. Adult schistosomes are blood dwelling trematode helminths which excrete eggs via the urine (*S*. *haematobium*) and faeces (*S*. *mansoni*, *S*. *japonicum*). Eggs that are not excreted can become trapped in human tissues causing a peri-ovular granulomatous inflammatory reaction [1], the main mechanism of disease in schistosomiasis. The largest burden of infection lies within sub-Saharan Africa although there is a significant burden of disease in other regions of Africa, the Middle East, Asia, South America [4], and more recently, cases from a focus in southern Europe [5]. In a review of UK travellers presenting with acute schistosomiasis [6], Lake Malawi was found to be the commonest site of exposure (53% of patients). As global travel increases, an awareness of the complications of this condition is vital.

Neuroschistosomiasis is a rare and severe complication of schistosomiasis triggered by the immune reaction to egg deposition in the central nervous system (CNS). It is almost entirely caused by *S*. *mansoni*, *S*. *haematobium* or *S*. *japonicum*. *S*. *mansoni* and *S*. *haematobium* usually affect the spinal cord [7,8,9,10,11] whereas *S*. *japonicum* usually causes intracranial disease [12]. Neuroschistosomiasis is estimated to affect between 1% and 4% of people with systemic schistosomal infections [4]. This is thought to be secondary to two possible mechanisms: 1) the adult Schistosoma worms lay eggs in the hepatic portal system that then travel through the valveless paravertebral veins of Batson (Batson’s plexus) to reach the lower spinal cord and 2) adult worms migrate to the CNS and produce eggs *in-situ* in the local venules [4]. Worldwide, 90% of cases of neuroschistosomiasis are estimated to occur in sub-Saharan Africa [8] although an increasing number of cases outside of endemic areas are being noted due to global travel.

The diagnosis of neuroschistosomiasis can be difficult. Along with a history consistent with the risk of exposure, observation of schistosomal ova in urine, stool or rectal biopsy, or positive serum schistosomal serology can support a diagnosis [1]. However, the absence of eggs in these samples does not exclude neuroschistosomiasis. Serology is valuable in making the diagnosis but it must be interpreted in the context of a travel history and any previous treatment. Cerebrospinal fluid (CSF) tends to indicate an inflammatory phenotype with elevated protein and white cell count, often with a lymphocyte predominance; eosinophils are estimated to be detectable in 50% of patients’ CSF [1]. Neuroschistosomiasis leads to characteristic changes on imaging which can be crucial in diagnosis. MRI findings typically include: enlargement of spinal cord (especially the lower cord and conus medullaris); thickening of spinal nerve roots (especially cauda equina); intermedullary T2 hyperintense and T1 hypointense signal reflecting oedema; and on post gadolinium T1 weighted images a heterogeneous multinodular pattern of contrast enhancement, thought to be related to ova induced granulomas within the spinal cord [1,4]. Spinal cord schistosomiasis is traditionally divided into three clinical forms: medullary (spinal cord predominant), myeloradicular (spinal cord and nerve root predominance), and a conus-cauda equina syndrome. However, patients may often present with either a combination of the above or evolve between these divisions during their disease progression. Spinal schistosomiasis typically presents as a lower cord syndrome of acute/subacute nature with typical symptoms of lower limb or back pain, leg weakness and sensory disturbance, and bladder, sphincter and erectile dysfunction [1,4]. A definitive diagnosis can only be made with histopathological sampling of CNS tissue though this is rarely done due to the risks involved [1].

The accepted criteria for confirmation of spinal cord schistosomiasis are as follows [1]:

1. Evidence of lower thoracic or upper lumbar neurological lesions clinically and on imaging
2. Evidence of infection with schistosomiasis through parasite isolation in tissue or serological techniques
3. Exclusion of other causes of myelitis.

Early treatment with praziquantel and high dose steroids leads to a reduction of neurological sequelae [9, 11–13]. There are no consensus guidelines or randomized controlled trials for the treatment of neuroschistosomiasis. Regimens combine anti-parasitics, corticosteroids and surgery in selected cases. Praziquantel is the anti-parasitic agent of choice for schistosomiasis with a reported cure rate of 70–90% [12]. Dosing regimens vary from 40–60 mg/kg/day given in divided doses, and treatment duration varies widely from 1–14 days in neuroschistosomiasis. Duration of steroid weaning is highly variable in reported cases [12]. Surgery is typically reserved for people with neuroschistosomiasis who have severe neurological symptoms and evidence of CSF flow obstruction [4].

We note that in non-endemic regions, neuroschistosomiasis is rare [1,4,8] and there is evidence that diagnosis can be delayed due to lack of clinical awareness of this condition. We present a series of patients with spinal cord schistosomiasis presenting to a national referral centre in the United Kingdom. We aim to provide an update on this condition to improve recognition and clinical management of patients with neuroschistosomiasis, with a focus on spinal schistosomiasis, the most common CNS complication.

## Methods

### Ethics statement

Ethical approval for this study was deemed not required according to University College London Hospitals policy. Signed patient consent forms were obtained from individual patients included within the case series.

### Study design

We performed a retrospective review of confirmed clinical cases of neuroschistosomiasis referred to a national referral centre for parasitic infections involving the central nervous system.

### Study setting

The Hospital of Tropical Diseases (HTD) works in partnership with the National Hospital for Neurology and Neurosurgery (NHNN) in providing a national referral centre for neuroschistosomiasis and other tropical neurological disorders including neurocysticercosis and hydatid disease. All cases were discussed at the regular Neuroparasitology Multidisciplinary Team Meeting (MDT). The Neuroparasitology MDT is based at the HTD, London. It receives referral cases nationally from the UK, often following discussion with specialist teams locally. The MDT includes neurology, neuroradiology and parasitology specialists with expertise in parasitic infections. Diagnosis is made based on clinical history, radiology and available parasitological results. Serum and CSF antibody detection ELISAs are performed in-house, in the national Parasitology Referral Laboratory, accredited by the national United Kingdom Accreditation Service.

### Data collection

Cases of neuroschistosomiasis discussed in the HDT Neuroparasitology MDT between January 2016 and January 2020 were reviewed. Electronic referral records were interrogated and patient demographic, clinical, laboratory, and radiological data included. Routine clinical information was collated and analysed, and pathology data was analysed for confirmatory testing.

## Results

### Case 1

**Fig 1:**
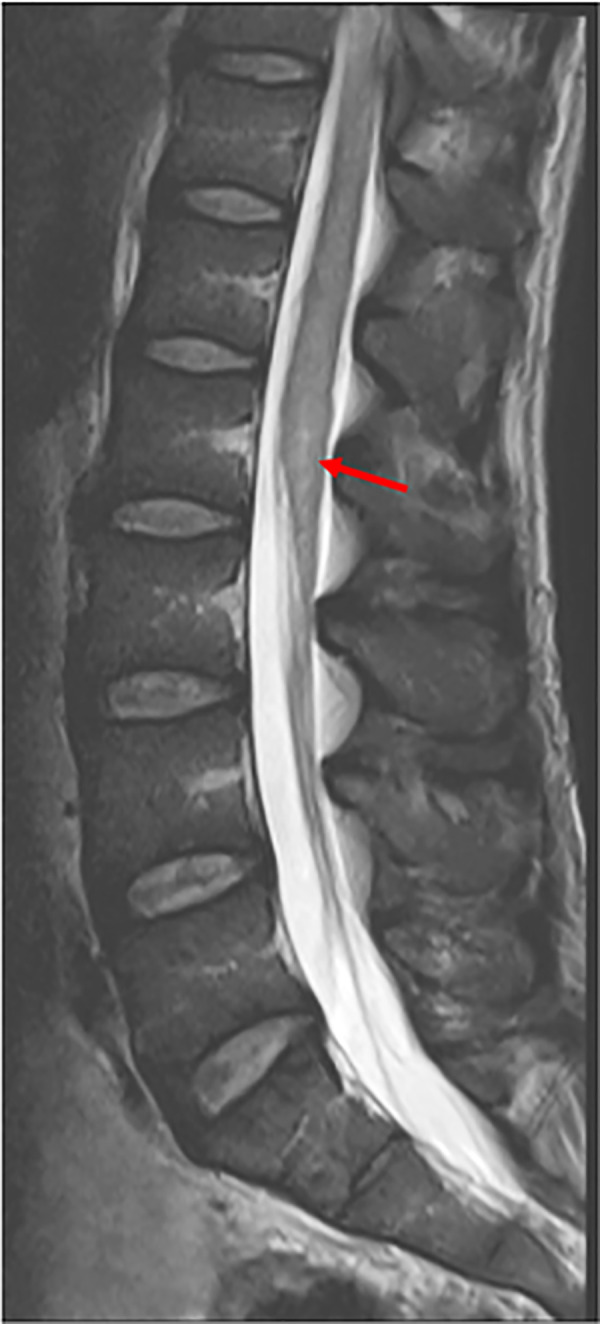
Initial MRI of patient 1 (locally reported as normal): showing subtle T2 hyperintense signal change (arrow) and mild swelling of conus medullaris.

A 50 year old male Nigerian clerical worker presented to UK health services in June 2016 with a short history of fever and back pain. He had returned from rural travel to Nigeria, where he had been undertaking farming work for 1 year; he reported swimming regularly in a local freshwater lake. On admission, his malaria film was positive for *Plasmodium falciparum* (0.1% parasitaemia) and he was treated with a 3 day course of atovaquone-proguanil combination therapy. During the first four days of his admission he continued to report back and leg pain. On more detailed questioning he revealed these symptoms had been ongoing 3 weeks prior to admission, with a 7 day history of constipation. On day 4 of admission he went into urinary retention and was unable to walk. He was noted to have a rising eosinophil count, increasing from normal on admission to 1.2x10^9/L on day 4. He underwent a lumbar spine MRI which was initially locally reported as normal but showed in retrospect high signal and swelling of the conus medullaris (Fig 1). He continued to deteriorate neurologically with flaccid paraplegia and was treated for presumed Guillain Barre Syndrome with intravenous immunoglobulin. He subsequently underwent a repeat MRI, reported as transverse myelitis of the thoracic spine from T4-T10. CSF analysis showed a CSF glucose within normal range at 4.2mg/dl, elevated CSF protein at 1.87g/L and a white cell count (WCC) of 12 leucocytes/mm^3^ (100% monocytes). The case was discussed with the UK Imported Fever Service who directed further investigation of the case. The discussion centred on the possibility of spinal schistosomiasis, or *Strongyloides* infection exacerbated by HTLV-1. HTLV-1 and strongyloides investigations were subsequently negative. The patient was discussed in the HTD Neuroparasitology MDT where the initial unenhanced MRI was felt to demonstrate a subtle conus lesion. The repeat whole spine MRI had nodular enhancement typical of spinal schistosomiasis. Urgent schistosomiasis serology was arranged by the HTD parasitology team and returned strongly positive on serum (ELISA optical density (OD) 1.132, cut off 0.26) and CSF (ELISA OD 1.139, cut off 0.26). Terminal urine was negative for ova. Treatment was commenced 16 days after presentation to UK medical services. The exact time from exposure to freshwater to symptom onset was unclear, as the patient was likely to have been exposed regularly during his year in Nigeria when swimming in a local lake. He completed a 5 day course of praziquantel under dexamethasone cover, which was tapered over the course of one year. He also received empirical treatment with ivermectin (15mg per day for 2 days) to cover the possibility of coincidental occult *Strongyloides* infection which might fulminate on steroid therapy.

Subsequent to this treatment, the patient received neurorehabilitation and his mobility improved and the patient was able to walk unaided at 2 months. The patient was given a second course of praziquantel in November 2017 as a precaution to ensure eradication of schistosomiasis, due to concerns regarding ongoing spasticity and bladder dysfunction. There was no history of re-exposure. Repeat schistosomal serology was negative the following year in October 2018 (OD 0.212, cut off 0.26). Despite this, the patient had persistent complications of neuroschistosomiasis including a broad-based gait, and ongoing urinary dysfunction which required botox detrusor injections and urinary sheath use at 3 year follow up. In addition, he reported ongoing lumbar back and leg pain which was managed with pregabalin and specialist pain team input. During the prolonged steroid treatment, the patient developed steroid induced insomnia, weight gain, and a heel ulcer thought to be related to steroid therapy.

### Case 2

**Fig 2:**
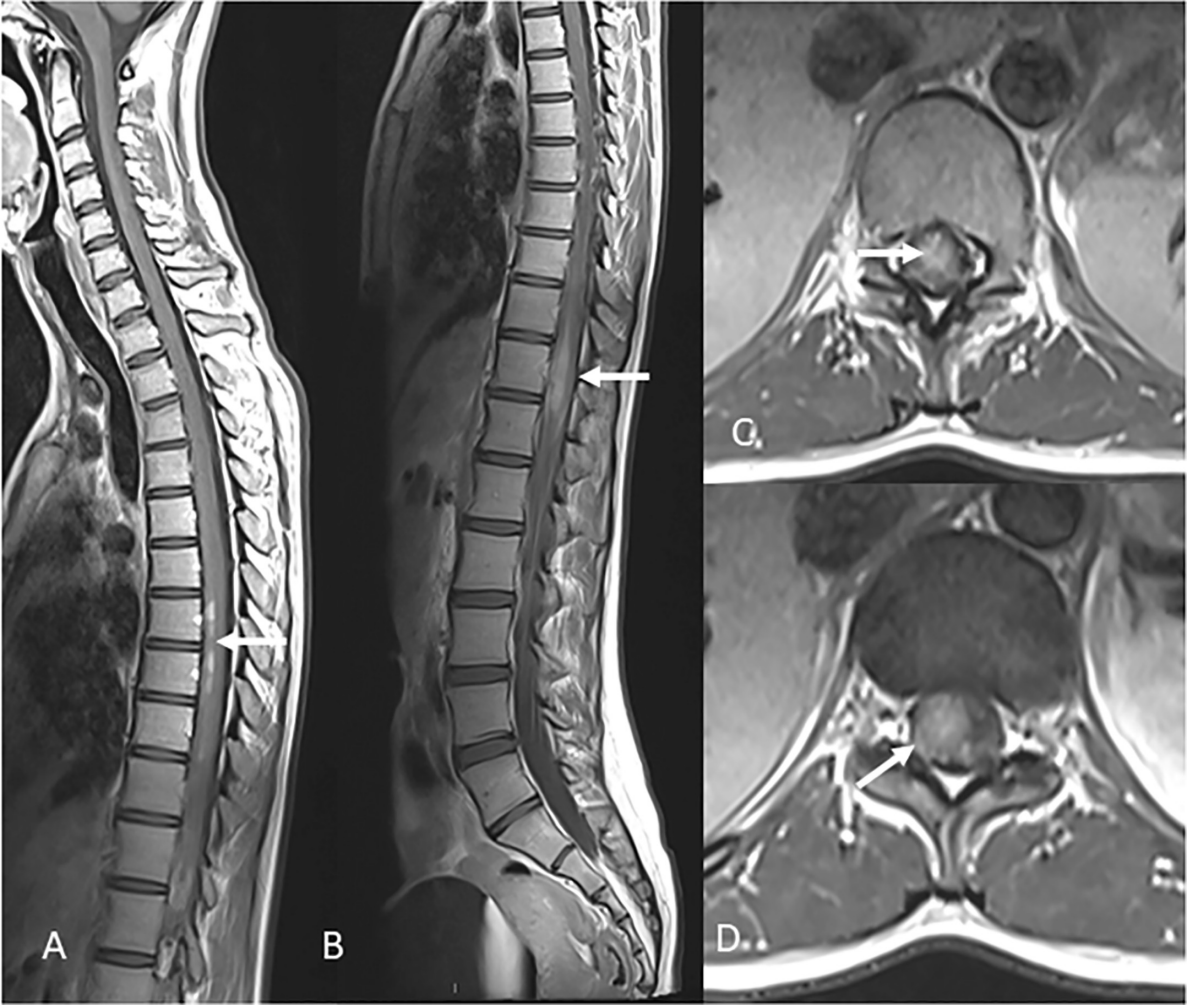
Sagittal (A and B, patient 2) and axial (C and D, patient 2) gadolinium-enhanced images of spine demonstrating pathological contrast enhancement at D7+D8 and D11+D12 levels (arrows).

A 21 year old female student presented to UK medical services on 1st June 2018. She presented with several months of fatigue, followed by an acute 3 day history of lower back pain and progressive leg weakness. On presentation, the patient had difficulty with micturition and defecation, and was unable to walk. She was initially diagnosed and treated for neuromyelitis optica with high dose steroids and plasma exchange. She was discharged to a rehabilitation unit and required walking aids to mobilise at this stage. At a subsequent clinical encounter the patient volunteered a travel history, recalling swimming in the Ugandan Nile in June 2017 and in Thailand in July 2017, prompting consideration of a tropical infection causing her symptoms. At this point, her case was discussed with the HTD Neuroparasitology MDT where a diagnosis of spinal schistosomiasis was felt likely, and appropriate investigations coordinated. Schistosomal serology returned positive on serum (OD 0.48, cut off 0.26) and CSF (OD 0.543, cut off 0.26). CSF microscopy revealed an elevated WCC of 297 leukocytes/mm^3^ which were predominantly lymphocytes (87%). The patient’s CSF protein was normal at 0.4g/L and CSF glucose 3.9 (no paired serum sample available). MRI spine demonstrated extensive T2 hyperintense signal change throughout the spinal cord which was most prominent in the lower thoracic cord and conus medullaris. Contrast enhanced imaging of the spine demonstrated two areas of pathological enhancement (D7 to D8 and at D11 to D12), (Fig 2) A diagnosis of neuroschistosomiasis was made and she was treated initially with 1 day (40mg/kg/day) in August 2018 followed by a 3 day course (40mg/kg/day) in September 2018 alongside a 6 month course of weaning steroids. She received a repeat course of praziquantel (20mg/kg TDS for 3 days) to cover the possibility of *S*. *japonicum* infection in October 2018. Following anti-schistosomal treatment her symptoms completely resolved and she was discharged from follow up. Time from exposure to freshwater in a schistosomiasis endemic region to presentation with symptoms was 11 months. Length of time from initial presentation to commencing appropriate anti-parasitic treatment was 74 days. Follow up MRI in January 2019 showed oedema within the spinal cord was much improved with no enhancement. No treatment related adverse events were described. She remains symptom free at the time of reporting.

**Fig 3:**
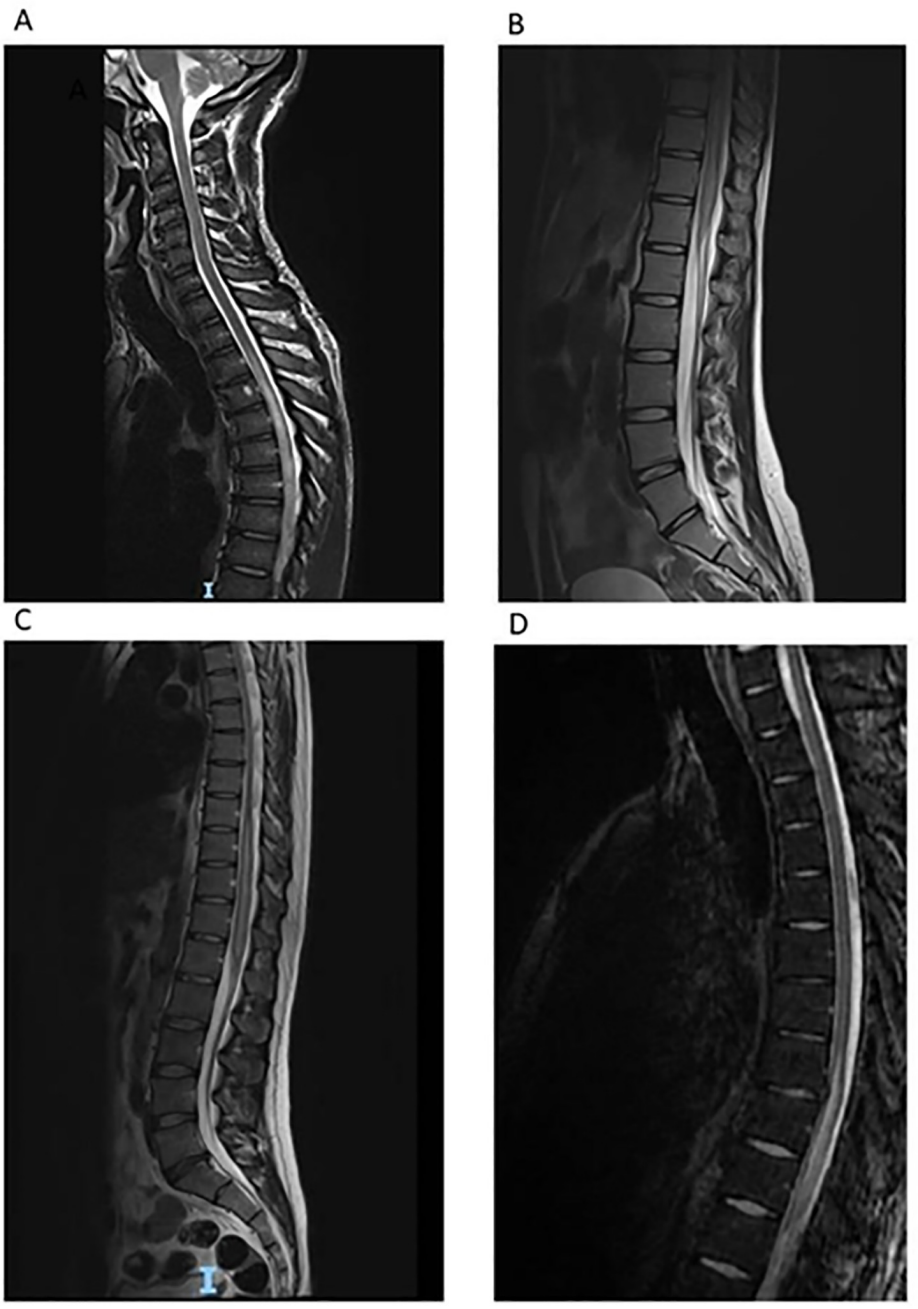
Sagittal T2 weighted MRIs in patients 1 (A), 2 (B), 3 (C) and 4 (D) showing intramedullary hyper intense signal, affecting the lower thoracic cord/conus medullaris in all patients with variable extension into mid thoracic cord.

Fig 3A and 3B detail further imaging findings for case 1 and 2 respectively.

### Case 3

A 31 year old male presented to UK medical services on 7th November 2018 with two weeks of lower limb pain, paraesthesia, and dysuria. His dysuria was initially treated as a urinary tract infection but failed to improve. Following worsening of his symptoms a lumbar puncture and MRI were performed. CSF microscopy showed elevated WCC of 297 (261 lymphocytes, 36 polymorphs); with elevated eosinophil count, a moderately raised protein level of 0.96g/dl and glucose within the normal range. He reported travel to Malawi in October 2017 during which he recalled swimming and undertaking water sports in Lake Malawi. MRI spine demonstrated high signal in the conus and signs of severe oedema up to the level of the thoracic spine (Fig 3C). There was contrast enhancement revealing multifocal cord lesions. He was initially treated with 3 days of intravenous methylprednisolone and plasma exchange for an inflammatory transverse myelitis at his local hospital. Routine bloods were normal and peripheral eosinophil counts were not raised during admission, ranging between 0.0–0.4x10^9/L. On 13th December 2018 he was discussed in the Neuroparasitology MDT which recommended treatment for neuroschistosomiasis based on imaging findings and epidemiological risk. He received praziquantel (60mg/kg/day for 3 days). He was subsequently found to have positive serum schistosomal serology (serum ELISA positive at 1.5, cut off 1.2; IgG confirmed on Western blot, *external laboratory result*); in-house CSF schistosomal serology was positive (OD 0.677, cut off 0.26). Length of time from exposure to freshwater in an endemic region to symptom onset was 12 months. Length of time from initial UK presentation to commencing appropriate anti-parasitic treatment was 31 days. He had a recurrence of worsening neurological symptoms following the first attempt to wean his steroids, but they were successfully tapered and stopped in July 2019. Steroid related side-effects were described including elevated blood glucose, transaminitis, Cushingoid facies, cystic acne, folliculitis, insomnia and anxiety. His dermatological side-effects warranted referral to tertiary care for dermatology input. At 18 month follow-up he had ongoing complications of neuroschistosomiasis including dysesthesias in his lower limbs and constipation requiring laxatives.

### Case 4

A 25 year old male pilot presented to UK medical services on 3rd of August 2019 with symptoms of pain and paraesthesia of both legs, and difficulty passing urine. He was found to be in urinary retention with a residual volume of 1 litre and was catheterised. He reported travel to Uganda in March 2017 on a military expedition during which he canoed from Lake Victoria along the White Nile and was in contact with river water. No advice on schistosomiasis prevention was given before the trip and no screening for schistosomiasis was undertaken after the trip. During his admission he was found to have a varying peripheral blood eosinophil count (range 0.0 to 0.6x10^9/L). MRI spine performed on 9th August 2019 showed high abnormal T2/STIR signal in the spinal cord from T7-T11 (Fig 3D). He was initially diagnosed and treated as a case of inflammatory transverse myelitis, receiving 5 days of intravenous methylprednisolone in August 2019. CSF analysis demonstrated an elevated protein of 0.7g/L and WCC of 132 (81% lymphocytes). CSF schistosomal ELISA was positive (OD 1.7, cut off 0.26). CSF glucose was not available at time of writing. Serum schistosomal serology performed at another laboratory was positive (ELISA positive at 8.8, cut off 1.2; IgG confirmed on Western blot). After discussion with the parasitology team at the HTD, he commenced a 3 day course of praziquantel (60mg/kg/day) in September 2019 alongside a 3 day course of 60mg prednisolone followed by a steroid wean over 66 days. The diagnosis was confirmed in the Neuroparasitology MDT. At 8 months’ follow up he requires ongoing intermittent self-catheterisation for improving but ongoing urinary retention. He retains reduced left ankle dorsiflexion (MRC grade 4+/5) and bilaterally absent ankle jerks. He requires the occasional use of a stick to aid walking. He has improved but ongoing subjectively altered sensation of bowel movements. The length of time from exposure to freshwater in a schistosomal endemic region and symptom onset was 29 months. Length of time from initial UK presentation to commencing appropriate anti-parasitic treatment was 44 days. No specific adverse effects from medication were reported for this patient.

### Summary of case series

**Fig 4:**
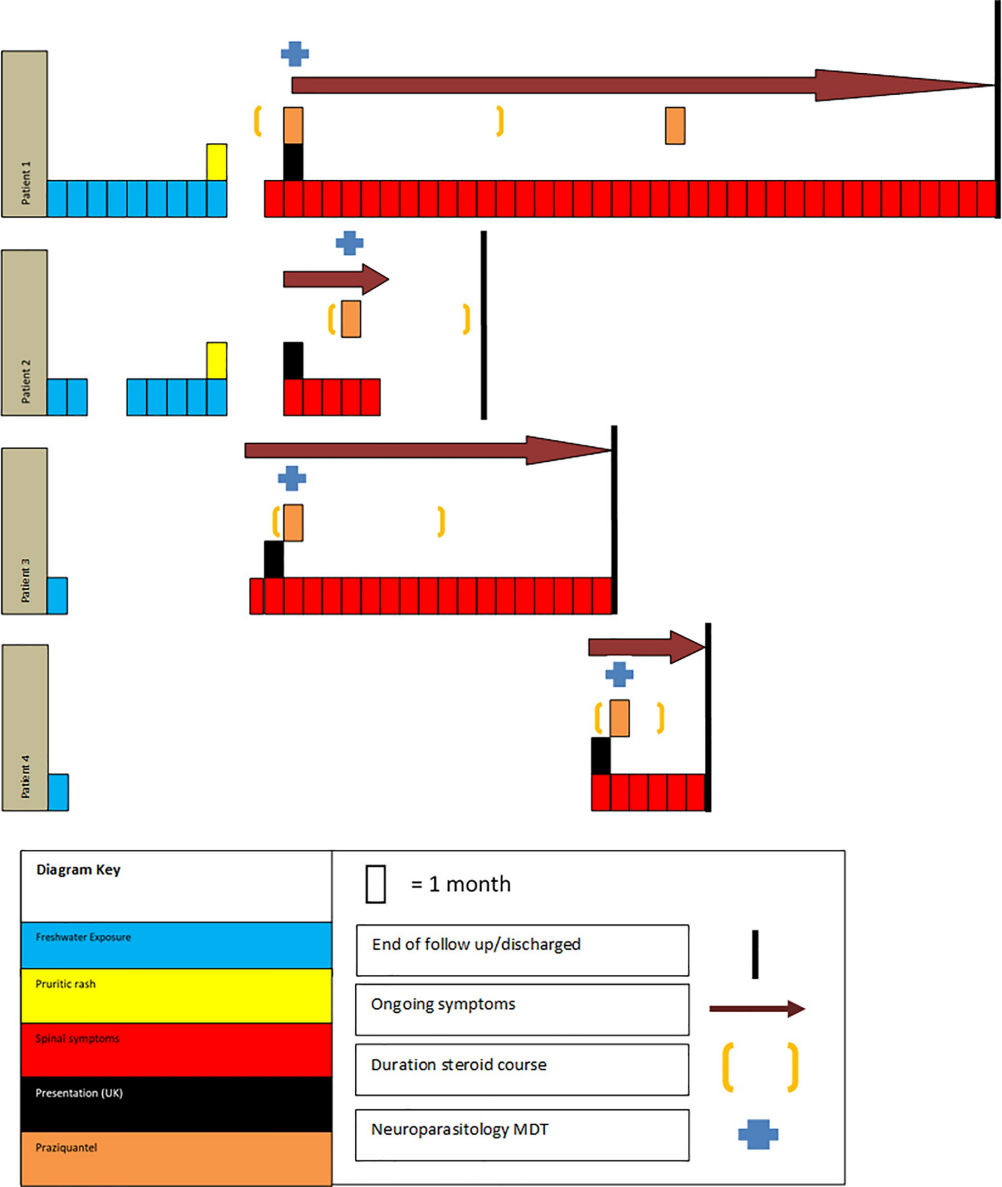
Diagram showing timing of exposure, symptom development, healthcare seeking, treatment and outcomes of neuroschistosomiasis.

Four cases of neuroschistosomiasis were referred to the Neuroparasitology MDT over a four year period (Table 1 and Fig 4). The median age at diagnosis was 28 (range 21 to 50) with three male patients. All patients had epidemiological risk factors for schistosomiasis based on travel history and freshwater exposure; two in Uganda (River Nile), one in Malawi and one in Nigeria. Mean time between travel from endemic regions and presentation was 12.7 months (range 0–29 months) (Fig 4). All patients presented with back pain, leg weakness, paraesthesia and urinary dysfunction. Two patients also presented with abnormal defecation. Of those, one had constipation and one had faecal incontinence. Mean time from first presentation to UK health services to commencement of praziquantel was 42.15 days (range 16–74 days) (Fig 4). Peripheral blood eosinophilia was detected in three out of four patients, however detection of schistosomal ova in stool or urine was reported in no patients; rectal snips were not performed. CSF lymphocytosis was present in all four patients. Diagnosis was confirmed with CSF serology for schistosomiasis in all cases. Radiology features included typical enhancement of the lower thoracic spine in three cases and enhancement of the conus in one patient. We note that in one case (patient 1) MRI findings were only noted to be typical for schistosomiasis on review by a neuroradiologist with expertise in parasitic neurological infection. All patients received a minimum of 3 days praziquantel and high dose steroids. Clinical features resolved fully in only one out of four patients.

**Table 1:** Clinical features of Neuroschistosomiasis cases.

Patient	Age	Sex	Country of exposure	Time since travel (months)	Presentation to anti parasitic treatment (days)	Blood eosinophilia	CSF WCC count (differential)	Serum schistosomiasis serology	CSF schistosomiasis ELISA	Anti-parasitic therapy (length)	Duration steroid wean	Outcome/ongoing morbidly
1	50	M	Nigeria	0	16	Yes	14 (100% monocytes)	Positive	Positive	Praziquantel (5 days)	One year	Urinary dysfunction, broad based gait, back and leg pain, steroid induced insomnia, weight gain and heel ulcer.
2	31	M	Malawi	11	36	No	37 (100% monocytes)	Positive	Positive	Praziquantel (3 days)	8 months	Constipation; leg pain; reduced sensation; steroids—cushingoid facies, cystic acne; recurrence on weaning steroids initially
3	21	F	Uganda	11	74	Yes	261 lymphocytosis, 36 polymorphs	Positive	Positive	Praziquantel (3 days)	6 months	Full recovery
4	25	M	Uganda	29	44	Yes	132 (81% lymphocytes)	Positive	Positive	Praziquantel (3 days)	66 days	Urinary dysfunction requiring ongoing intermittent self-catheterisation;

## Discussion

Spinal cord involvement is a rare but severe complication of schistosomiasis. We report four cases of spinal schistosomiasis diagnosed in the UK. All four cases had relevant travel histories and presented with spinal cord syndromes. This series provides the most detailed description of a series of spinal schistosomiasis diagnosed in the UK, and highlights the delayed time from presentation to medical services to commencement of effective treatment, an important prognostic factor for long term morbidity.

The major clinical features found in our cases were found to be lower limb weakness, altered sensation, sphincter dysfunction (most commonly urinary retention), and back and leg pain. Lambertucci et al [13], based in a schistosomal endemic area, have previously described twenty-three patients with neuroschistosomiasis of whom 70% were unable to walk unaided at presentation, in keeping with the severity described in our cohort. In our series only one patient made a complete neurological recovery, with the remaining three patients demonstrating some improvement following anti-parasitic and steroid treatment. Once again, this is similar to previously reported outcomes; Lambertucci et al reported a 21.7% (5/23) rate of full neurological recovery in their cohort [13], highlighting the severity of neuroschistosomiasis.

Since the diagnosis of neuroschistosomiasis was not initially considered, the first patient was treated as a case of Guillain Barre Syndrome until the exposure history and MRI scans were reviewed. The other three patients were diagnosed as cases of inflammatory myelitis and treated with high dose steroids and in one case intravenous immunoglobulin. Despite incorrect diagnoses in these cases, fortunately steroids are part of management for spinal schistosomiasis. However it is clear that misdiagnosis can result in the administration of unnecessary treatments and particularly late prescription of anti-parasitic agents alongside steroids, which is likely to be suboptimal.

Significant delay was observed in diagnosis and treatment of neuroschistosomiasis in our patients. There is concern that this is likely to lead to poorer outcomes. Often specialist guidance from the Neuroparasitology MDT at the Hospital for Tropical Diseases was sought after relevant exposure history was elicited, or volunteered by patients. In our patient with the shortest time of presentation-to-treatment (16 days), travel history was elicited early by the attending neurologist, who was then able to seek advice from specialist infectious diseases services. Furthermore, the maximum time from travel to an endemic region and presentation (29 months), illustrates the potential pitfalls in taking only a recent travel history. Therefore, we advocate the importance of a full travel history, including freshwater exposure, in all patients presenting with spinal cord syndromes.

Serum and CSF schistosomal serology was of particular use for diagnosis. Both testing modalities were positive in all our cases. We found peripheral eosinophilia to be present in three out of four patients prior to treatment. All our patients had a reactive CSF but the presence or absence of a CSF eosinophilia was only reported in one patient. Unfortunately, in cases confirmed by histopathology following biopsy of spinal lesions, testing of blood and CSF for anti-schistosomal antibodies is infrequently reported [13], leading to an as yet poorly defined specificity and sensitivity of immunoassays in confirmed spinal schistosomiasis. Our report adds value to the available literature by suggesting that in cases of spinal schistosomiasis, high quality CSF serology assays should be positive. Lambertucci et al [13] reported 90% of patients to have elevated CSF protein levels, and elevated eosinophilis in 40% of cases sampled in an endemic area. They also found eggs in stool, urine or positive peripheral blood serology were non-specific and not sufficient for diagnosis of neuroschistosomiasis. However, that would not be the case in returning travellers normally resident in locations where schistosomiasis is not transmitted.

Detection of schistosomal ova in stool, urine or rectal biopsies is not an essential requirement for diagnosis, not least because eggs from ectopic worm pairs in Batson’s plexus are unable to reach the intestine or urinary tract. There is heterogeneity within the literature with regard to what constitutes a confirmed diagnosis of neuroschistosomiasis.

We recommend that all patients with a consistent travel history for schistosomal exposure and neurological symptoms have schistosomal serology from serum and CSF sent urgently to avoid a delay in diagnosis. The reference laboratory should be telephoned in advance to ensure that samples are prioritised for testing. Additionally, a raised peripheral eosinophilia in the context of neurological symptoms should prompt clinical suspicion of a parasitic infection involving the CNS.

Our patients were diagnosed in a non-endemic setting where clinical exposure to neuroschistosomiasis is rare. We found discussion in the specialist Neuroparasitology MDT provided or confirmed a diagnosis in all four patients, prompted testing in three of four cases, and the MDT team directed management in all four patients. In the case of patient 3, his treatment was in fact directed prior to serology results on the basis of clinical history, epidemiology and typical MRI findings. The typical imaging findings of spinal schistosomiasis are demonstrated well by our four cases; T2 hyperintense signal change in the lower thoracic cord and conus medullaris, heterogeneous nodular enhancement; and oedema and swelling of the cord. These imaging findings are often misdiagnosed as tumours and inflammatory transverse myelitis [7]. The differential diagnosis for such findings also includes spinal cord oedema secondary to a dural arteriovenous fistula.

Subtle changes recognised in a specialist MDT with the presence of an appropriate epidemiology history can expedite diagnosis; this is most clearly demonstrated in the case of patient 1, with an initial MRI spine reported locally as normal but with typical features of spinal schistosomiasis identified in the Neuroparasitology MDT. This highlights the need for potential cases of neuroschistosomiasis to be referred early to an expert MDT in non-endemic settings where clinician exposure to such conditions locally may be sparse. Furthermore, early discussion in a specialist MDT may avoid the need for spinal biopsy in cases of diagnostic difficulty by pointing to a diagnosis of neuroschistosomiasis.

In summary, neuroschistosomiasis can cause severe and permanent disability. Prompt diagnosis and treatment relies on taking of an effective travel history and can be improved by discussion of cases with specialists in the diagnosis and management of neuroparasitic infections.

## References

[1] Ferrari TC, Moreira PR. Neuroschistosomiasis: clinical symptoms and pathogenesis. Lancet Neurol. 2011 9;10(9):853–64. 10.1016/S1474-4422(11)70170-3 . 21849166

[2] Chitsulo L, Engels D, Montresor A, Savioli L. The global status of schistosomiasis and its control. Acta Trop. 2000 10 23;77(1):41–51. 10.1016/s0001-706x(00)00122-4 ; PMCID: PMC5633072. 10996119 PMC5633072

[3] Ross AG, Bartley PB, Sleigh AC, Olds GR, Li Y, Williams GM et al Schistosomiasis. N Engl J Med. 2002 4 18;346(16):1212–20. 10.1056/NEJMra012396 . 11961151

[4] Vale TC, de Sousa-Pereira SR, Ribas JG, Lambertucci JR. Neuroschistosomiasis mansoni: literature review and guidelines. Neurologist. 2012 11;18(6):333–42. 10.1097/NRL.0b013e3182704d1e . 23114664

[5] Boissier J, Grech-Angelini S, Webster BL, Allienne JF, Huyse T, Mas-Coma S et al Outbreak of urogenital schistosomiasis in Corsica (France): an epidemiological case study. Lancet Infect Dis. 2016 8;16(8):971–9. 10.1016/S1473-3099(16)00175-4 Epub 2016 May 17. . 27197551

[6] Logan S, Armstrong M, Moore E, Nebbia G, Jarvis J, Suvari M et al Acute schistosomiasis in travelers: 14 years’ experience at the Hospital for Tropical Diseases, London. Am J Trop Med Hyg. 2013 6;88(6):1032–4. 10.4269/ajtmh.12-0646 Epub 2013 Mar 25. ; PMCID: PMC3752798. 23530076 PMC3752798

[7] Kim AH, Maher CO, Smith ER. Lumbar intramedullary spinal schistosomiasis presenting as progressive paraparesis: case report. Neurosurgery. 2006 5;58(5):E996; discussion E996. 10.1227/01.NEU.0000210223.25400.C7 . 16639309

[8] Joshi TN, Yamazaki MK, Zhao H, Becker D. Spinal schistosomiasis: differential diagnosis for acute paraparesis in a U.S. resident. J Spinal Cord Med. 2010;33(3):256–60. 10.1080/10790268.2010.11689703 ; PMCID: PMC2920119. 20737799 PMC2920119

[9] Pittella JEH. 2003 Neuroschistosomiasis. In: Misra UD, Kalita J, Shakir RA, eds. Tropical neurology. Georgetown: Landes Bioscience, 2003: 300–24 13

[10] Carod-Artal FJ. Neuroschistosomiasis. Expert Rev Anti Infect Ther. 2010 11;8(11):1307–18. 10.1586/eri.10.111 . 21073294

[11] Ferrari TC. Spinal cord schistosomiasis. A report of 2 cases and review emphasizing clinical aspects. Medicine. 1999 5;78(3):176–190. 10.1097/00005792-199905000-00004 10352649

[12] Clerinx J, van Gompel A, Lynen L, Ceulemans B. Early neuroschistosomiasis complicating Katayama syndrome. Emerg Infect Dis. 2006 9;12(9):1465–6. 10.3201/eid1209.060113 ; PMCID: PMC3294743. 17073109 PMC3294743

[13] Vandack Nobre, Silva Luciana CS, Ribas João G, Rayes Abdunnabi, Serufo JC, Lana-Peixoto MA et al Schistosomal myeloradiculopathy due to Schistosoma mansoni: report on 23 cases. Mem. Inst. Oswaldo Cruz [Internet]. 2001 9 [cited 2020 Oct 15]; 96 (Suppl): 137–141. 10.1590/S0074-02762001000900020 11586439

